# Component Identification and Functional Analysis of Outer Membrane Vesicles Released by *Avibacterium paragallinarum*

**DOI:** 10.3389/fmicb.2020.518060

**Published:** 2020-09-25

**Authors:** Chen Mei, Ai-hua Sun, Patrick J. Blackall, Hong Xian, Shu-fang Li, Yu-mei Gong, Hong-jun Wang

**Affiliations:** ^1^Beijing Key Laboratory for Prevention and Control of Infectious Diseases in Livestock and Poultry, Institute of Animal Husbandry and Veterinary Medicine, Beijing Municipal Academy of Agriculture and Forestry, Beijing, China; ^2^Queensland Alliance for Agriculture and Food Innovation, The University of Queensland, St Lucia, QLD, Australia

**Keywords:** *A. paragallinarum*, outer membrane vesicles, mass spectrometry, nucleic acids, functional analysis, immunogenicity

## Abstract

*Avibacterium paragallinarum*, the causative agent of infectious coryza, is known to release outer membrane vesicles (OMVs). In the present study, we investigated the composition, bioactivities, and functional properties of the OMVs of *A. paragallinarum*. Following extraction and purification, the OMVs were observed to be spherical in shape, with diameters ranging from 20 to 300 nm. The vesicles contained endotoxin as well as genomic DNA. The molecular weights of the OMV-contained protein fragments were mostly concentrated at 65 and 15 kDa. The components of the OMV proteins were mainly various functional enzymes (e.g., ATP-dependent RNA helicase), structural components (e.g., streptomycin B receptor and membrane protein), and some hypothetical proteins with unknown functions. The expression levels of inflammation-related factors, such as interleukin (IL)-2, IL-6, IL-1β, IL-10, and inducible nitric oxide synthase (iNOs), were significantly upregulated in chicken macrophage cells HD11 incubated with OMVs. Serum IgG antibodies were measured after two intramuscular injections of an OMV-based vaccine into specific pathogen-free (SPF) chickens. The vaccinated chickens were then challenged by *A. paragallinarum* of homologous and heterologous serovars. It was noted that the vaccinated chickens produced immunoglobulin G (IgG) antibodies against *A. paragallinarum*. The OMVs conferred an acceptable level of protection (70%), defined as an absence of colonization and of clinical signs, against the homologous strain (serovar A), while the cross-protection against heterologous challenge with serovars B and C was much weaker. However, the OMVS did provide significant protection against clinical signs for all three serovars. Overall, this study laid a foundation for further unraveling the functional roles of OMVs released by *A. paragallinarum*.

## Introduction

Infectious coryza is an acute respiratory disease of poultry that causes significant economic losses by inducing growth retardation and impairing egg production. The disease is caused by *Avibacterium paragallinarum*, a Gram-negative bacterium that inflicts damage to the nasal and respiratory epithelium (Blackall and Soriano-Vargas, 2020). The continuing problem of outbreaks of infectious coryza, including in North America (Crispo et al., 2018) and Europe (Heuvelink et al., 2018), highlights the importance of unraveling the interaction between *A. paragallinarum* and the host immune response.

The release of outer membrane vesicles (OMVs) by Gram-negative bacteria has been observed and extensively studied over the past decades (Jan, 2017), including *Haemophilus influenzae* (Deich and Hoyer, 1982; Roier et al., 2016) and *A. paragallinarum* (Ramon Rocha et al., 2006) in the family *Pasteurellaceae*. OMVs are ubiquitously produced both *in vitro* and *in vivo* during an infection (Roier et al., 2016) and play a crucial role in host-microbe interactions (Turnbull et al., 2016). OMVs can transport a wide variety of chemically diverse cargoes (Schooling and Beveridge, 2006), such as lipids and lipopolysaccharides (LPS; Marisa Heredia et al., 2016), membrane-embedded and membrane-associated proteins, nucleic acids, and other small molecules (Kulp and Kuehn, 2010). Additionally, OMVs are involved in intracellular communication, microbial virulence, and the regulation of host immune responses (Tashiro et al., 2010). Furthermore, OMVs have been evaluated as vaccine antigens targeting *H. influenzae*, *Gallibacterium anatis*, *Pasteurella multocida*, *Mannheimia haemolytican*, and *Actinobacillus pleuropneumoniae* (Roier et al., 2012, 2013; Pors et al., 2016; Antenucci et al., 2018). However, little is known about the immunogenic potential of OMVs derived from *A. paragallinarum*.

In order to understand the characteristics of *A. paragallinarum* OMVs and their relationship with host immunity, a study on the components, functions, and biological activities of the OMVs is reported here. The immunogenicity of the OMVs was tested *via* chicken vaccination and challenge experiment.

## Materials and Methods

### Bacterial Strains and Cell Line

Three isolates (Hp8, BJ, and 668) of *A. paragallinarum* obtained from chickens in China and previously identified to be Page serovar A, B, and C, respectively (Xing et al., 1986; Chen et al., 1993; Zhang et al., 2003) were used in this study.

Chicken macrophage HD11 cell line was derived from NEWGAINBIO (HD11, CL1337, NEWGAINBIO, Wuxi, China).

### Chickens

Specific pathogen-free (SPF) chickens (42 days old) were obtained from the Experimental Animal House of the China Pharmaceutical University (Beijing, China). They were given free access to pelleted feed (Beijing Cooperation Medical and Pharmaceutical Company, Beijing, China). All animal work in this study met the minimum standards of animal welfare as described in the International Guiding Principles for Biomedical Research involving Animals (at https://grants.nih.gov/grants/olaw/Guiding_Principles_2012.pdf). The handling of chickens was in accordance with the Guidelines of Animal Care and Use Committee of the Institute (IACUC) under the approval of Institute of Animal Husbandry and Veterinary Medicine (Permit number: 2014-05). All efforts were made to avoid animal suffering and to improve the quality of life of the birds.

### Isolation and Purification of OMVs

*A. paragallinarum* strain hp8 (Page serovar A) was grown in 10 ml of tryptic soy broth (TSB, BD, United States) supplemented with 10% chicken serum and 2.5% nicotinamide adenine dinucleotide (NAD) at 180 rpm for 15 h at 37°C. The culture was then transferred to 500 ml of supplemented TSB and incubated for 12 h. Subsequently, the whole bacterial cells were removed by centrifugation at 10,000 × *g* for 10 min at 4°C, and the collected supernatant was filtered using Stericup filters (pore size, 0.45 μm; Millipore Corporation, Billerica, MA, United States) to remove floating cell debris. The filtered supernatant was centrifuged by ultracentrifugation in a 45 Ti rotor (Beckman Coulter, Krefeld, Germany) at 235,000 × *g* for 3 h at 4°C to yield a crude OMV preparation. Then, the crude OMVs were resuspended in 2 ml HEPES buffer (50 mM HEPES, 150 mM NaCl, pH 6.8) containing 45% w/v iodixanol. Highly purified OMVs were prepared using OptiPrep™ (60% w/v iodixanol in water, Fresenius kabi Norge AS, Oslo, Norway) density gradient centrifugation. The crude OMVs preparation was placed in an Ultra Clear™, 14 ml, 14 × 95 mm tube (Beckman Coulter, Krefeld, Germany). A discontinuous iodixanol gradient was achieved by layering successive 2 ml volumes of HEPES buffer containing 40%, then 35, 30, 25, 20, and 15% w/v iodixanol. After centrifugation at 156,000 × *g* for 180 min at 4°C (using a SW40 Ti rotor installed in an Optima L-80XP Ultracentrifuge, Beckman-Coulter, Krefeld, Germany), the visible enriched fractions in the various layers of the density gradient were collected by pipette (in a sequence from top to bottom). Gradient fractions containing purified OMVs were identified by protein analysis using the bicinchoninic acid (BCA) protein assay kit (Thermo Scientific, United States) according to the manufacturer’s instructions. After that, the highly purified OMVs were diluted with sodium chloride-Tris-ethylenediaminetetraacetic acid (EDTA) buffer (STE; Santa Cruz, CA, United States) and centrifuged at 156,000 × *g* for 120 min at 4°C (using a SW40 Ti rotor, Beckman-Coulter, Krefeld, Germany). The pellet was collected and resuspended in 1 ml phosphate-buffered saline (PBS) as the final OMV preparation and stored at −20°C.

### Transmission Electron Microscopy

Formvar-coated copper grids were floated on 20 μl droplets of purified membrane vesicle solutions/and or purified OMVs with *A. paragallinarum* [a single colony, grown on tryptic soy agar (TSA, BD, United States) supplemented with 10% chicken serum and 2.5% NAD for 15 h at 37°C in candle jar] for 20 min. The grids were rinsed in water and then floated on 1% uranyl acetate for 30 s. After washing again with water, the grids were imaged on a Philips CM 100 transmission electron microscopy (TEM) at an accelerating voltage of 80 kV.

### Characterization of DNA Present in OMVs

To eliminate membrane-associated nucleic acids, an issue reported by Ramon Rocha et al. (2006), in the OMV preparation, 1 U/μl of DNAse I (Thermo Fisher Scientific, CA, United States) was added to the preparation, and the mixture was incubated for 10 min at 37°C. Next, OMVs were pelleted by centrifuging at 15,000 × *g* for 15 min, and the supernatant was removed by micropipette aspiration. OMVs were disrupted by incubating at 70°C for 10 min with 400 μl of VL buffer [40 mM Tris-acetate, pH 7.8, 20 mM sodium acetate, 1 mM EDTA, 1% sodium dodecyl sulfate (SDS; w/v)]. The mixture was cooled to room temperature in a water bath for 3 min. To remove OMV debris from the suspension, 150 ml of 5 M sodium chloride was added, mixed by inverting the tube 30 times, and centrifuged (15,000 × *g*, 4°C, 10 min).

DNA was extracted from the DNase-treated OMVs by a commercial kit (Bacterial Genomic DNA Extraction Kit, Solarbio, China). A PCR that targeted the bacterial 16S ribosomal RNA (rRNA) gene was performed using the primer pair 27F (AGAGTTTGATCCTGGCTCAG) and 1492R (GGTTACCTTGTTACGACTT; Garcia-Sanchez et al., 2014). After amplification, products were purified using a commercial kit (Invitrogen PCR product purification kit, Life Technologies, United States) and were directly sequenced using an ABI PRISM BigDye Terminator V3.1 kit (Applied Biosystems, CA, United States). The sequences were analyzed and edited using Sequencing Analysis V5.2 Software (Applied Biosystems, CA, United States) and compared with already published sequences in the GenBank, NCBI, using the BLAST tool.

The purified DNA from OMVs was also sent for whole genome sequencing (Allwegene Technologies, China) on the Illumina HiSeq 4000 PE150 platform with massively parallel sequencing (MPS) Illumina Technology. Illumina PCR adapter reads and low quality reads from the paired-end and mate-pair library were filtered by a quality control step that used the provided compiling pipeline. All good-quality paired reads were assembled using the SOAP *de novo* software with at least 100-fold coverage^1^ into a number of scaffolds. Then, the filtered reads were subjected to gap closing.

### Measurement of OMVs Endotoxin

The kinetic chromogenic limulus amebocyte lysate (LAL) assay (Kinetic-QCL; Lonza) for endotoxin measurement was performed as previously described (Thorne, 2000). Briefly, 10-fold dilutions, prepared in pyrogen-free water, of the OMV preparations were made in depyrogenated borosilicate tubes. Twofold dilutions of the control standard endotoxin/LPS (*Escherichia coli* O55:B5, Sigma, SC, United States) were assayed to create a 12-point standard curve ranging from 0.0244 to 50.0 endotoxin units (EU)/ml. The preparations and controls were assayed in 96-well microplates, (Corning) and the absorbance change was measured at 405 nm every 30 s for 90 min using a microplate reader (Spectra Max 384 Plus, Molecular Devices, with Softmax PRO 4.0). All samples assayed by the LAL assay were diluted to yield at least four separate dose points that fell within the controlled standard endotoxin (CSE) curve. Quality assurance measures for the LAL assay were as previously described (Thorne et al., 2015).

### Protein SDS-PAGE

Detection of proteins from OMVs on gel electrophoresis was carried out according to the manufacturer’s instructions. The 20-μl OMV sample was loaded on to a 12% sodium dodecyl sulfate-polyacrylamide gel electrophoresis (SDS-PAGE) gel. After electrophoresis, the gel was transferred to a staining solution containing Coomassie Blue Stain G-250. The gel was then destained with 15% (v/v) methanol and 10% (v/v) acetic acid.

### Mass Spectrometry

The bands of interest from two hp8 OMV SDS-PAGE gel lanes (corresponding to 65 and 15 kDa) were excised, pooled, and cut into 1-ml cubes. Then, the samples were subjected to mass spectrometric analysis at the Beijing Genomics Company by using a Q Exactive mass spectrometer (Thermo Scientific, CA, United States). Only doubly and triply charged ions, with an intensity of >15 counts/s, were selected for tandem mass spectrometry (MS/MS) analysis. Data acquisition and processing were performed using MassLynx version 4.0 software (Micromass, United Kingdom). All samples were measured in triplicate.

### Cells Culture, Treatment, and RNA Extraction

Chicken macrophage cells (HD11) were cultured in Dulbecco’s modified Eagle’s medium (DMEM) high glucose (Invitrogen Corporation, MA, United States) supplemented with 10% fetal serum, 100 U/ml streptomycin, and 100 mg/ml penicillin at 37°C in an atmosphere of 5% CO_2_. The medium was changed three times per week, according to a standard cell culture protocol. After reaching at least 85% confluence, cells were suspended in a complete growth medium. Trypan blue (100 μl of a 0.1% solution) was added into 100 μl of the cell suspension and gently mixed. Cells were then counted with a hemocytometer, as per the manufacturer’s instructions. The cells, adjusted to 2 × 10^6^/ml, were plated in 25-cm^2^ flasks (Corning, NY, United States) at 5 ml/flask.

HD11 cells were treated with 5 μg/ml OMVs (protein concentration) for 24 and 48 h. In addition, the control HD11 cells were treated with 1 μg/μl LPS (*E. coli* O55:B5, Sigma, United States) for 24 and 48 h as well. Total RNA was isolated from OMV or LPS-treated cells with TRIzol reagent (Invitrogen). RNA concentration was measured with NanoDrop One (Thermo Fisher Scientific, WI, United States) using the optical density ratio at 260 and 280 nm (OD260/OD280). Complementary DNA (cDNA) was synthesized by RevertAid First Strand cDNA Synthesis Kit (Thermo Scientific, CA, United States) according to the manufacturer’s instructions. The cDNA samples were stored at −20°C for subsequent qRT-PCR.

### Detection of Inflammation-Related Genes

A quantitative real-time PCR (qRT-PCR) assay was carried out using a LightCycler® 96 SW 1.1 instrument (Roche, Germany). The reaction mixture was composed of SYBR Premix Ex Taq II (Takara, China), the relevant specific primer set (Table 1), and DNA template. β-actin was used as a housekeeping gene (De Boever et al., 2008). Quantitative PCR was performed with ABI ViiA7 Real-Time PCR System (Applied Biosystems, Carlsbad, CA, United States). The amplification program used was as follows: an initial denaturation step at 95°C for 10 min, followed by 30 cycles of denaturation at 95°C for 30 s, annealing at 60°C for 1 min and extension at 72°C for 1 min, and a final extension step at 72°C for 8 min. The results were analyzed by the CT method using the onboard data assist software (version 3.0, Applied Biosystems/Life Technologies). The data were normalized to the β-actin gene expression.

**Table 1:** List of primer sequences^a^ used for quantitative real-time PCR (qRT-PCR) analysis.

Gene	Prime sequence (5'–3')	NCBI reference sequence
IL-1β	F: TGAGCACAGGACAGTGGACG R: CTCCGCAGCAGAATGGTCAT	NM_204524.1
IL-6	F: AATCCCTCCTCGCCTTTCTG R: GCCCTCACGGTCTTCTCCAT	NM_204628.1
IL-2	F: GGAGTGCACGGAGCAAACTC R: TCCGGTGTGATTTACTCCCG	NM_204153.1
iNOS	F: GCCAGAATTAGTGGACGGCC R: GCTTGCCCAATAGGACCTT	NM_204961.1
IL-10	F: CCTTTGGCTGCCACACTGTG R: GCCCATGCTCACCTGATGAC	NM_001004414.2
β-actin	F: CACAGATCATGTTTGAGACCTT R: CATCACAATACCAGTGGTACG	L08165.1

a All primers were designed in this study except the primers for β-actin, which were from De Boever et al. (2008).

### Hemagglutination

The hemagglutination (HA) activity of OMVs was determined using a standard HA microtiter assay. Briefly, twofold dilutions of OMVs in 25 μl volumes of PBS were prepared in duplicate in a U-bottomed microtiter plate. Thereafter, 25 μl of either 1% fresh or glutaraldehyde-fixed chicken red blood cells was added to each well containing OMVs plus the control wells that contained 25 μl of diluent only. The plates were incubated at room temperature for 30–60 min, and red blood cells agglutination was detected by direct visual inspection.

### OMV Vaccine Evaluation

A mixture of OMVs and MONTANIDE™ ISA 71 VG adjuvants (SEPPIC, Shanghai, China) was emulsified at a 3:7 mass ratio (antigen:adjuvant). The vaccine, termed V-OMVs-A, contained 100 μg/ml of hp8 OMVs (protein concentration). The 42-day-old SPF chickens were randomly divided into a V-OMVs-A vaccinated group and a PBS control group (*n* = 30 in each group). A 0.5-ml volume of V-OMVs-A was injected into chicken by the breast muscle route in the vaccinated group. The second injection was administered at 2 weeks after the first dose. The control birds were inoculated with PBS.

Four weeks after the second immunization, all chickens from each group were challenged with one of the three serovars of *A. paragallinarum* (10 chickens for each serovar) – hp8 (5 × 10^5^ CFUs/0.2 ml), BJ (2 × 10^5^ CFUs/0.2 ml), and 668 (5 × 10^5^ CFUs/0.2 ml), by intranasal inoculation. Clinical signs of infectious coryza, such as mucous exudate from the nostrils, sneezing, swelling of the infraorbital sinuses, facial edema, and conjunctivitis, were recorded from the second to the eighth day postchallenge. All birds were monitored daily, and an individual score was given based on clinical signs: 0, active with no adverse clinical sign; 1, mild facial swelling and or nasal discharge; 2, moderate facial swelling and or nasal discharge, slightly weak with dropped wings weak; and 3, severe facial swelling and or nasal discharge, unresponsive with eyes closed. All chickens were then humanely euthanized, and swabs, taken from both infraorbital sinuses, were cultured on blood agar with a *Staphylococcus epidermidis* nurse colony (Soriano et al., 2004). The inoculated plates were incubated at 37°C with 5% CO_2_. The presence of satellitic colonies, indicating the recovery of the challenge strain of *A. paragallinarum*, was recorded after overnight incubation. Protection was defined as the absence of clinical signs of infectious coryza and a failure to reisolate *A. paragallinarum*.

### Determination of Serum IgG

Four weeks after the second immunization, serum samples were collected from all chickens, and antibody titers against OMVs were tested by ELISA. A 96-well microplate (Corning) was coated with 1 μg/well of sonicated bacterial antigen prepared from hp8 and diluted with a carbonate/bicarbonate buffer (pH 9.6). The coated plates were incubated overnight at 4°C. After blocking with 5% skimmed milk for 2 h (at 37°C), a 100-μl aliquot of the serum sample (diluted 1:100 in PBS) was added into the 96-well microplate and incubated at 37°C for 1 h. Then, 100 μl of horseradish peroxidase conjugated goat antichicken IgG (IgG-HRP; Abcam, Cambridge, United States) was added as a secondary antibody, followed by incubation at 37°C for 1 h. Finally, the microplate was read at 450 nm using an EON microplate spectrophotometer (Biotek, VT, United States).

### Statistical Analysis

Data in the results were analyzed by the independent sample *t*-test and one-way ANOVA using GraphPad Prism software (GraphPad Software, Inc., San Diego, CA, United States). All results were presented as mean ± standard deviation (SD). *p* < 0.05 were regarded as statistically significant.

## Results

### Ultrastructure of the OMVs

The OMVs were found to be both closely associated with bacterial cells as well as distinctly separated from the cells of *A. paragallinarum*. The OMVs were spherical in shape and had membranous structures with diameters varying between 20 and 300 nm (Supplementary Figure S1).

### Detection of Nucleic Acids in OMVs

OMVs contained DNA at an estimated concentration of 124 ng/μl. The 16S rDNA PCR of the OMVs produced a product that was similar in size (1,398 bp) to the product obtained when using *A. paragallinarum* hp8 as template (data not shown). In addition, the 16S rRNA gene sequence of the OMV preparation (accession number MK806690) and multiple sequences of the 16S rDNA of *A. paragallinarum* were up to 99.86% identity with multiple sequences of *A. paragallinarum* 16S rDNA in GenBank.

The total DNA extracted from the OMVs was examined by agarose gel electrophoresis (data not shown). The genomic sequences of the OMVs of *A. paragallinarum* strain hp8 (BioSample accession: SAMN12734518) were composed of 184 contigs for 2,357,467 bp with a 40.99% G + C content. The largest contig was 98,601 bp, and the minimum contig was 263 bp. Automatic gene prediction was performed using GeneMarkS.^2^ In total, 2,382 protein-encoding genes were predicted, with a coding percentage of 86.76%. The average gene length was 859 bp. In addition, the genes were searched against the Kyoto Encyclopedia of Genes and Genomes (KEGG; Kanehisa et al., 2004) and Clusters of Orthologous Groups (COG; Tatusov et al., 2003) databases to annotate the gene description. Based on the analysis, the functions of 1,448 genes (60.8% of the total of 2,382 genes) were predicted, and 2,016 genes (84.6%) were assigned into 23 clusters of COG functions. As well, one predicted copy of both the 16S and the 23S rRNA genes and six copies of 5S rRNA gene were detected using rRNAmmer software (Lagesen et al., 2007).

### Analysis of Endotoxin and Proteins in the OMVs

The level of endotoxin in OMVs was 211.2 EU/ml. The total concentration of OMV proteins was found to be 1.632 mg/ml. The OMV proteins appeared to consist of proteins of approximately 65 and 15 kDa by SDS-PAGE (data not shown).

Mass spectrometric analysis showed that all proteins with a confidence score of ≥21 from both the 65‐ and 15-kDa bands were associated with *A. paragallinarum* bacterial proteins (Tables 2 and 3). The components of the OMV proteins were mainly various functional enzymes (e.g., ATP-dependent RNA helicase), structural components (e.g., streptomycin B receptor and membrane protein), and some hypothetical proteins with unknown functions.

**Table 2:** Protein components of the outer membrane vesicles (OMVs) of *Avibacterium paragallinarum* at 65 kDa.

Number	Score	Name of proteins^*^
1	37	ATP-dependent RNA helicase HrpA
2	34	Alanine:cation symporter family protein
3	32	Hypothetical protein
4	32	Lysine-tRNA ligase
5	31	Phosphoglycerate kinase
6	30	HlyD family secretion protein
7	26	Formate dehydrogenase accessory sulfurtransferase FdhD
8	25	RNA polymerase sigma factor RpoH
9	24	Type I restriction enzyme R protein
10	23	Hypothetical protein Z012_06385
11	23	Hypothetical protein
12	22	Putative acyltransferase
13	21	Hypothetical protein Z012_06020

* All named proteins were from *A. paragallinarum* with the putative acyltransferase protein being linked to strain JF4211.

**Table 3:** Protein components of the OMVs of *A. paragallinarum* at 15 kDa.

Number	Score	Name of proteins^*^
1	112	Ferrioxamine B receptor
2	83	Hypothetical protein
3	71	Molecular chaperone DnaK
4	41	Lysine-tRNA ligase
5	37	Hypothetical protein
6	32	Thermonuclease family protein
7	30	Bacteriophage replication protein A
8	28	DNA-protecting protein DprA
9	26	DUF927 domain-containing protein
10	26	Membrane protein
11	25	Hypothetical protein Z012_06385
12	23	YggW family oxidoreductase
13	23	Hypothetical protein
14	23	Hypothetical protein Z012_06020
15	23	Hypothetical protein
16	23	Copper-translocating P-type ATPase
17	22	Formate dehydrogenase accessory sulfurtransferase FdhD
18	22	Metal ABC transporter substrate-binding protein
19	21	Cytochrome c nitrite reductase subunit NrfD
20	21	Putative 50S ribosomal protein L20
21	21	Putative acyltransferase

* All named proteins were from *A. paragallinarum* with the putative 50S ribosomal and putative acyltransferase proteins being linked to strain JF4211.

### OMVs Promote the Expression of Inflammation-Related Genes in HD11 Cells

**Figure 1:**
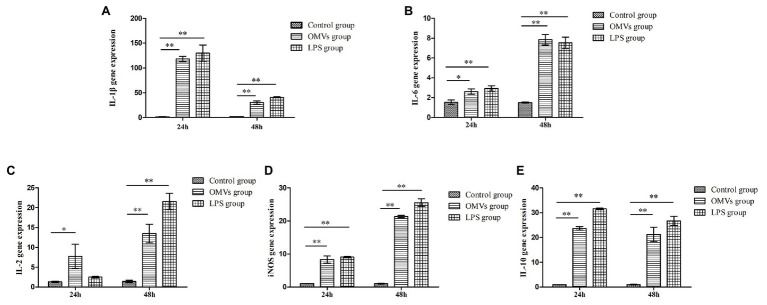
The expression levels of inflammation-related genes in HD11 cells. The expression levels of five inflammation-related genes in HD11were carried out with qRT-PCR assay, after promoted by OMVs. The results were calculated by 2^−ΔΔCt^ method. At 24 h, compared to the control group, the expression levels of **(A)** IL-1β, **(E)** IL-10, and **(D)** inducible nitric oxide synthase (iNOS) were markedly higher (*p* < 0.01) in the cells exposed to OMV and lipopolysaccharides (LPS), while the expression levels of **(B)** IL-6 and **(C)** IL-2 were considerably increased (*p* < 0.05) in OMV-exposed cells. At 48 h, all the five genes expressed significantly higher (*p* < 0.01) in OMV‐ or LPS-exposed group compared to the control group. Moreover, the expression level of **(E)** IL-10 and **(A)** IL-lβ had significantly decreased at 48 h, compared to 24 h. The results are expressed as mean ± SEM (different letters indicate statistical difference among groups analyzed by one-way ANOVA with *post hoc* Tukey test; ^**^p < 0.01; ^*^p < 0.05; *n* = 3).

The 2^−ΔΔCt^ method was used to calculate the levels of inflammation-related genes expression. At 24 h, compared to the control group, the expression levels of IL-1β, IL-10, and inducible nitric oxide synthase (iNOS) were remarkably higher (*p* < 0.01) in the cells exposed to OMV and LPS, while the expression levels of IL-6 and IL-2 were considerably increased (*p* < 0.05) in OMV-exposed cells (Figure 1). At 48 h, all five genes were expressed at significantly higher levels (*p* < 0.01) in the OMV‐ or LPS-exposed groups compared to the control group. Moreover, the expression level of IL-10 and IL-lβ had significantly decreased at 48 h compared to 24 h.

### Hemagglutination

No HA activity against either fresh or glutaraldehyde-fixed chicken red blood cells could be detected with the OMV sample.

### V-OMVs-A Vaccine Trial

**Figure 2:**
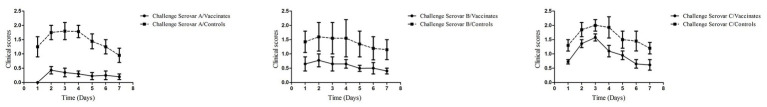
Clinical scores observed in the vaccination-challenge trial. Four weeks after the second immunization, all chickens from each group were challenged with one of the three serovars of *A. paragallinarum*, by intranasal inoculation. Clinical signs of infectious coryza were recorded for 7 days postchallenge. Data were given as mean values of clinical signs in each group. Within each serovar, the vaccinated/challenge group has a lower clinical signs score at each observation day than the control group.

The results of the vaccine trial demonstrated that all chickens in the control groups exhibited typical clinical symptoms of infectious coryza (Table 4; Figure 2). In the V-OMV-A group challenged with hp8, only three chickens showed clinical signs with the same three chickens also yielding *A. paragallinarum*, i.e., a protection rate of 70%. As well, the daily mean clinical signs was significantly lower for the vaccinated chickens compared with the controls (Figure 2). The protection in OMV-vaccinated birds challenged with either BJ or 668 was much lower, 10 and 30%, respectively. However, for all three serovars, the clinical signs at all daily observations were significantly lower in the vaccinates than in the controls (Figure 2). These results suggest that the immunogenicity of vesicles is providing a degree of cross-serovar protection (in terms of clinical signs) but that the ability to significantly lower colonization is serovar specific.

**Table 4:** Results of challenge test of OMV-vaccinated chickens with *A. paragallinarum* isolates of different serovars.

Vaccine	Challenge strain (serovar)	No. of ELISA positives	Cumulative number of sick chickens^*^	*A. paragallinarum* reisolation rate	Protection rate^**^
V-OMVs-A	Hp8 (A)	10	3	30%	70%
	BJ (B)	10	9	90%	10%
	668 (C)	10	7	70%	30%
Control	Hp8 (A)	0	10	100%	0
	BJ (B)	0	10	100%	0
	668 (C)	0	10	100%	0

* The statistical analysis of clinical scores are shown in Figure 2.

** The protection rate for the vaccinated birds challenged with serovar A was significantly different (*p* < 0.05) from the controls challenged with serovar A. No significant difference in terms of protection existed between the vaccinated and the controls for serovars B and C.

### Chicken Serum IgG Antibody Levels

**Figure 3:**
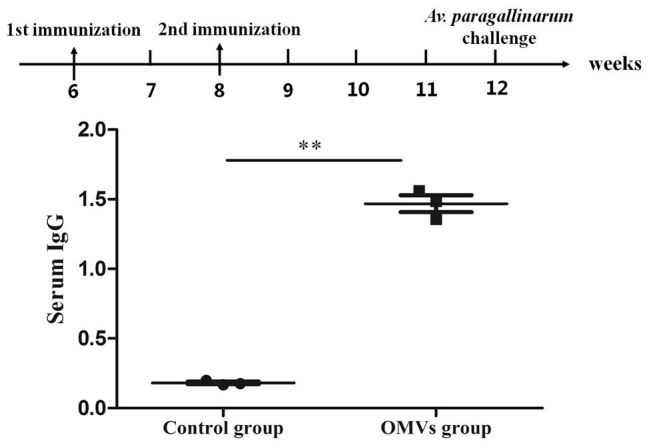
Serum immunoglobulin G (IgG) response in specific pathogen-free (SPF) chickens against OMVs. The IgG response is shown as OD650, calculated from an ELISA assay (sera dilution 1:100) against sonicated bacterial antigen. Chickens in the OMV group were given two doses of V-OMVs-A vaccine prepared from OMVs of *A. paragallinarum* serovar A strain hp8. Chickens in the control group were given two doses of phosphate-buffered saline (PBS). Four weeks after the second vaccination, sera were collected, and antibody titers were tested by ELISA. Asterisks indicate statistical difference between OMVs group and control (^**^p < 0.01).

Serum IgG antibody were measured in OMV-vaccinated and control groups at 4 weeks after the secondary immunization (Figure 3). The IgG antibody level in the OMV-vaccinated group was significantly higher (*p* < 0.01) than that in control group. These results indicate that OMVs can stimulate strong humoral immunity in vaccinated chickens.

## Discussion

The existing commercial vaccines against *A. paragallinarum* are typically inactivated multivalent whole-cell bacterins and provide only serovar-specific protection (Blackall and Soriano-Vargas, 2020). *A. paragallinarum* is a fastidious, slow-growing organism. Hence, producing a whole-cell vaccine, which often contains a Page serovar A, B, and C strain, requires a complex process that includes growth of the seed strains, viable counting of each strain, and often concentration of each strain to meet the concentration needed for each antigen. Further, these whole-cell vaccines occasionally cause side effects such as face swelling or local necrotic lesions at the injection site (Sakamoto et al., 2013). Therefore, to develop a safe, universal, effective, and low-cost vaccine is a key priority.

OMVs are naturally nonreplicating, highly immunogenic spherical nanoparticles derived from Gram-negative bacteria (Ho et al., 2015; Cecil et al., 2019). As OMVs from pathogenic bacteria have been successfully used as vaccines (Gerritzen et al., 2017), there is interest in the OMVs of *A. paragallinarum* as potential vaccine candidates.

In this work, most of the OMVs observed were spherical with a diameter that ranged from 20 to 300 nm. These results were similar to those previously reported by Ramon Rocha et al. (2006) who reported vesicle diameters that ranged from 25 to 300 nm. While a few of OMVs appeared as collapsed bubbles that were distributed around the bacteria, this is well recognized as being an artifact of the use of TEM (Pascucci and Scattini, 2020). Future studies on the OMVs of *A. paragallinarum* should involve additional characterization techniques such as light scattering properties (e.g., by high-resolution flow cytometry) as recommended by Théry et al. (2018).

The present study confirmed that *A. paragallinarum* OMVs are composed of proteins, LPS, and nucleic acids. Nucleic acids have been detected in OMVs from a range of bacteria and include both luminal and surface-associated DNA as reviewed by Jan (2017). Indeed, OMVs of *H. influenzae* were able to bind double-stranded DNA in a nuclease-resistant, salt-stable form (Deich and Hoyer, 1982). Using treatment with DNAse to eliminate external DNA and followed by disruption of OMVs, nucleic acids (approximately 12 kb in size) were detected inside the *A. paragallinarum* OMVs by Ramon Rocha et al. (2006). This prior work and current results suggest that the DNAs (i.e., the 16S rRNA gene and chromosomal DNA) detected from highly purified OMVs were luminal associated.

Short read sequencing of the OMV genomic DNA resulted in 184 fragments with a total length of 2.36 Mb, which is similar to the reported genome size of *A. paragallinarum*, between 2.33 and 2.87 Mb approximately, recorded in National Center for Biotechnology Information (NCBI).^3^ This raises the possibility that the genomic material detected in the OMVs may actually be the entire chromosomal DNA as reported for other bacteria (Jan, 2017). According to a previous report, OMV-associated DNA and proteins function as a source of carbon and nitrogen during the bacterial growth (Jan, 2017). As well, OMV-mediated transfer of genes coding for antibiotic resistance, virulence, and metabolic traits has been described in Gram-negative and Gram-positive bacteria (Domingues and Nielsen, 2017). Further characterization of the DNA content of *A. paragallinarum* OMVs and the biological roles of that DNA is required.

In this study, the protein components of *A. paragallinarum* OMVs were identified by mass spectrometry. We found that OMV protein components consisted of a mixture of membrane proteins, ATP-dependent RNA helicase, alanine:cation symporter family protein, phosphoglycerate kinase, etc. It is worth noting that these proteins play important biological roles in OMV-host cell interactions, leading to cell activation (Gagnon et al., 2006), cytokine secretion (Patel et al., 2013), and/or apoptotic cell death (Xia et al., 2017). As well, numerous putative proteins were identified with no functional labeling, which await further experimental exploration.

An earlier study on the OMVs of *A. paragallinarum* reported the presence of putative repeats-in-toxin (RTX) proteins (Ramon Rocha et al., 2006). The putative toxins were detected by immunoblotting (Ramon Rocha et al., 2006), which may explain why our study, based on direct SDS-PAGE examination and mass spectrometry, failed to detect similar proteins. In another difference, we failed to detect HA activity in our OMV preparation, an activity that was detected by Ramon Rocha et al. (2006) who detected this activity on a concentrated OMV preparation. It is possible that differences in methods and in OMV concentrations may explain the differences between our study and that of Ramon Rocha et al. (2006).

The OMVs of *Acinetobacter baumannii* interact with macrophages to induce the secretion of both pro‐ and anti-inflammatory cytokines, leading to uncontrolled chronic inflammation (Ahmad et al., 2019). It has been suggested that *A. paragallinarum* may have an immunomodulatory potential that influences host defense and inflammation (Paudel et al., 2017). Our results suggest that it is possible that the OMVs of *A. paragallinarum* involved in such influence on host responses. We examined five inflammation-related genes to determine whether host inflammation defenses were being stimulated and found that their expression levels in HD11 cells exposed either to OMVs or to LPS were indeed upregulated (Figure 1). It would seem likely that this stimulation of inflammatory markers by OMVs is due to the LPS component that we have detected.

Consistent with the earlier report of *Neisseria meningitidis* (Tavano et al., 2009), our results demonstrated that the OMVs of *A. paragallinarum* could upregulate the expression levels of IL-1β, IL-2, IL-6, IL-10, and iNOS, which indicate the activation of macrophages and existence of inflammatory process.

It has been previously demonstrated that proteins contained in *A. paragallinarum* OMVs were recognized by sera of chickens experimentally infected with, or vaccinated against, infectious coryza (Ramon Rocha et al., 2006). In the current study, we have shown that serum IgG levels were significantly higher in chickens vaccinated with OMVs compared to the control group, implying that OMVs can stimulate humoral immunity in chickens. The results of the challenge test showed that all vaccinated/challenge groups have significantly lower clinical signs scores at all daily observations than the control groups. However, immunization by OMVs only conferred a significant level of protection, defined as an absence of clinical signs and colonization, against hp8 (serovar A) and not against serovars B and C. This is probably related to the different components of antigen domains of the OMVs released by different serovars of *A. paragallinarum*. The ability to provide significant protection against clinical signs across all three serovars is a very promising finding that suggests that investigations to further improve this level of cross-serovar immunogenicity would be worthwhile. In particular, as the economic impact of infectious coryza is mainly a drop in egg production (Blackall and Soriano-Vargas, 2020), future work should include an examination of whether OMVs can protect against egg drops across the three Page serovars.

Preliminary *in vivo* testing of serovar A OMV-based vaccine did not show much protection to other two serovars. It is no wonder, as although OMVs possess various biological functions, they generally exhibit an antigenic pattern similar to the one found in the host bacterial outer membrane (Antenucci et al., 2018). Previous studies have shown that HMTp210, a 210-kDa outer-membrane protein (OMP) is the major protective antigen of *A. paragallinarum*, but its expression level was found very low. Nonetheless, the 1.6-kb hypervariable region (known as region 2) of HMTp210, which is serovar specific, encoding the most immunogenic antigen, has been demonstrated to produce effective serovar-specific protection (Sakamoto et al., 2013).

In follow-up experiments, we need to explore the biological effect of the individual components of the OMVs, RNA, protein, and LPS – using *in vivo* trials. This future work should compare the different subcomponents of the OMVs, seek nontoxic, protective immunogens, and determine their protective effects with different adjuvants and dose levels. Future vaccination-challenge trials should also focus on the ability of the OMVS to reduce the egg drop associated with infectious coryza outbreaks.

In summary, OMVs are involved in the pathogenesis of *A. paragallinarum* by secreting and delivering virulence factors and thus triggering both humoral and cell-mediated immunity. The preliminary study showed that *A. paragallinarum* OMVs, as a novel candidate vaccine, elicited a serovar-specific immune response against infectious coryza in chickens.

## Data Availability Statement

The datasets generated for this study can be found in the NCBI: accession number MK806690, NCBI: BioSample accession: SAMN12734518, the reference genome data of Av. paragallinarum, recorded in NCBI. (https://www.ncbi.nlm.nih.gov/genome/genomes/3481?).

## Ethics Statement

The animal study was reviewed and approved by Animal Care and Use Committee of the institute (IACUC) under the approval of Institute of Animal Husbandry and Veterinary Medicine (Permit number: 2014-05).

## Author Contributions

H-jW designed the experiments. CM, A-hS, HX, S-fL, and Y-mG performed the experiments. PB, HX, and H-jW analyzed the results. H-jW, PB, and CM wrote the paper. All authors contributed to the article and approved the submitted version.

### Conflict of Interest

The authors declare that the research was conducted in the absence of any commercial or financial relationships that could be construed as a potential conflict of interest.

## Footnotes

^1^http://soap.genomics.org.cn/soapdenovo.html

^2^http://topaz.gatech.edu/GeneMark/genemarks.cgi

^3^https://www.ncbi.nlm.nih.gov/genome/genomes/3481?
