# Supplementary material for: Component Identification and Functional Analysis of Outer Membrane Vesicles Released by Avibacterium paragallinarum
Source: Front Microbiol. 2020 Sep 25;11:518060. doi: 10.3389/fmicb.2020.518060 (PMC7545073; doi:10.3389/fmicb.2020.518060)
Supplement: Supplementary file 1 [file Data_Sheet_1.pdf]

Supplementary Figure

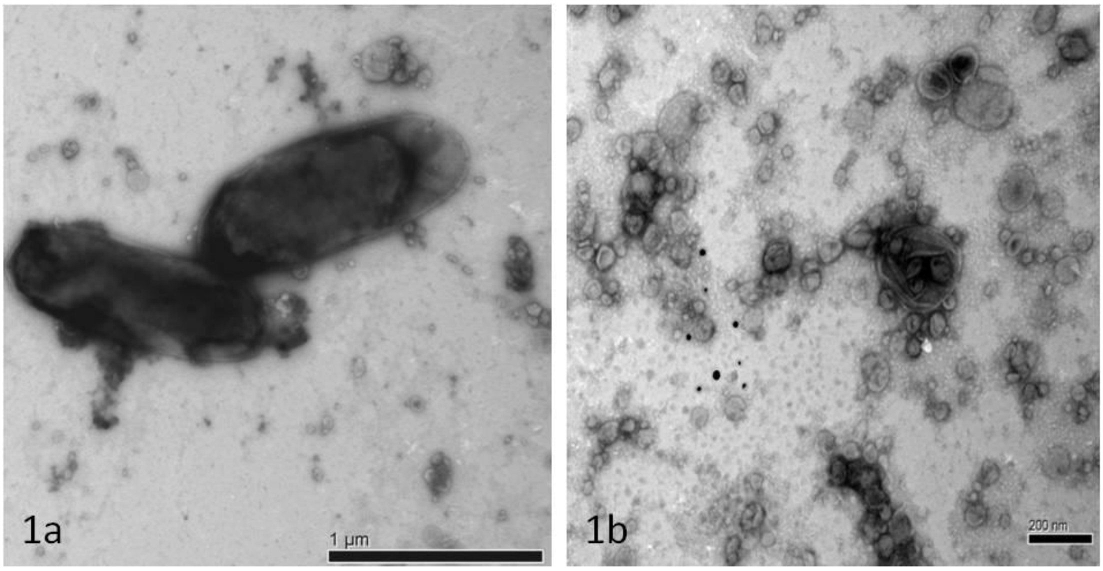

Figure S1. Transmission electron microscopy images: (1a) outer membrane vesicles (OMVs) with *Av. paragallinarum*, bar indicates 1 μm; (1b) purified OMVs, bar indicates 0.2 μm.
